# *Serendipita indica* mitigates drought-triggered oxidative burst in trifoliate orange by stimulating antioxidant defense systems

**DOI:** 10.3389/fpls.2023.1247342

**Published:** 2023-10-04

**Authors:** Yu Wang, Jin-Li Cao, Abeer Hashem, Elsayed Fathi Abd_Allah, Qiang-Sheng Wu

**Affiliations:** ^1^ College of Horticulture and Gardening, Yangtze University, Jingzhou, Hubei, China; ^2^ Botany and Microbiology Department, College of Science, King Saud University, Riyadh, Saudi Arabia; ^3^ Plant Production Department, College of Food and Agricultural Sciences, King Saud University, Riyadh, Saudi Arabia

**Keywords:** antioxidation, citrus, endophytic fungus, gas exchange, oxidative damage

## Abstract

Soil drought is detrimental to plant growth worldwide, particularly by triggering reactive oxygen species (ROS) burst. *Serendipita indica* (*Si*), a culturable root-associated endophytic fungus, can assist host plants in dealing with abiotic stresses; however, it is unknown whether and how *Si* impacts the drought tolerance of citrus plants. To unravel the effects and roles of *Si* on drought-stressed plants, trifoliate orange (*Poncirus trifoliata* L. Raf.; a citrus rootstock) seedlings were inoculated with *Si* and exposed to soil drought, and growth, gas exchange, ROS levels, antioxidant defense systems, and expression of genes encoding antioxidant enzymes and fatty acid desaturases in leaves were measured. Soil drought suppressed plant biomass, whereas *Si* inoculation significantly increased plant biomass (10.29%-22.47%) and shoot/root ratio (21.78%-24.68%) under ample water and drought conditions, accompanied by improved net photosynthetic rate (105.71%), water use efficiency (115.29%), chlorophyll index (55.34%), and nitrogen balance index (63.84%) by *Si* inoculation under soil drought. Soil drought triggered an increase in leaf hydrogen peroxide and superoxide anion levels, while *Si* inoculation significantly reduced these ROS levels under soil drought, resulting in lower membrane lipid peroxidation with respect to malondialdehyde changes. Furthermore, *Si*-inoculated seedlings under soil drought had distinctly higher levels of ascorbate and glutathione, as well as catalase, peroxidase, and glutathione peroxidase activities, compared with no-*Si*-inoculated seedlings. *Si* inoculation increased the expression of leaf *PtFAD2*, *PtFAD6*, *PtΔ9*, *PtΔ15*, *PtFe-SOD*, *PtCu/Zn-SOD*, *PtPOD*, and *PtCAT1* genes under both ample water and soil drought conditions. Overall, *Si*-inoculated trifoliate orange plants maintained a low oxidative burst in leaves under drought, which was associated with stimulation of antioxidant defense systems. Therefore, *Si* has great potential as a biostimulant in enhancing drought tolerance in plants, particularly citrus.

## Introduction

Drought stress (DS) is a frequent environmental factor that has a detrimental impact on plant physiological activities and morphological performance, such as lowering leaf gas exchange, slowing plant growth, and overproduction of reactive oxygen species (ROS) (Ahluwalia et al., 2021). ROS are highly reactive and toxic by-products of photosynthesis and photorespiration processes in plants, and the excess ROS causes oxidative damage to various macromolecules, thereby limiting plant growth and development (Ilyas et al., 2021; Tyagi et al., 2022a). Superoxide anion ( ${\text{O}}_{2}^{\cdot -}$ ) and hydrogen peroxide (H_2_O_2_) are the two most prevalent ROS induced by DS in plants (Miller et al., 2010). Plants also possess antioxidant defense systems to scavenge ROS, where antioxidant enzymes include superoxide dismutase (SOD), catalase (CAT), peroxidase (POD), and others, and non-enzymatic antioxidants include ascorbic acid (AsA), glutathione (GSH), carotenoids, and tocopherols (Mukarram et al., 2021). As a result, uncovering changes in antioxidant defense systems could clarify the drought-resistant potential of plants.

Citrus is the most widely grown fruit crop in the world (Addi et al., 2022). Because of its poor root hairs, trifoliate orange (*Poncirus trifoliata* L. Raf.), a common citrus rootstock, relies heavily on extraradical hyphae of arbuscular mycorrhizae in roots for water and nutrient uptake from the soil (Ortas, 2012). Symbiotic associations between arbuscular mycorrhizal fungi (AMF) and plants are prevalent, with AMF providing water and mineral nutrients to the host and the host providing carbohydrates to the fungal partner (Prasad et al., 2008; Tyagi et al., 2022b). Earlier studies have demonstrated that AMF could enhance drought tolerance in citrus, and the underlying mechanism is associated with mycorrhizal improvement of root structure and physiological activities, as well as stressed gene expression activation (Marulanda et al., 2007; Yaghoubian et al., 2014; Cheng et al., 2021; Liu et al., 2022; Wang et al., 2023). However, the application of AMF in the citrus field is limited because it cannot be cultured *in vitro* on a large scale without host plants. As a result, selecting an effective culturable endophytic fungus with functions similar to AMF has become a pressing problem in citriculture.

*Serendipita indica* (formerly *Piriformospora indica*) (*Si*) is a culturable endophytic fungus that can colonize a variety of host roots, including citrus (Varma et al., 2012; Yang et al., 2021a). *Si* possesses AMF-like characteristics (Mensah et al., 2020) and was isolated from an Indian desert (Verma et al., 1998), suggesting that it may be drought-tolerant. Earlier studies had reported significant increases in biomass and sustained growth in barley (*Hordeum vulgare*) and Arabidopsis (*Arabidopsis thaliana*) after inoculation with *Si* under drought (Sherameti et al., 2008; Ghaffari et al., 2019). Proteomics demonstrated that the colonization of *Si* raised photosynthesis-related protein levels in drought-stressed host plants (Ghaffari et al., 2019). *Si* colonization in cabbage (*Brassica campestris*) decreased leaf malondialdehyde (MDA) levels under DS, and several antioxidant enzyme activities were upregulated within 24 h (Sun et al., 2010). After *Si* inoculation, wheat (*Triticum aestivum*), eggplant (*Solanum melongena*), and walnut (*Juglans regia*) decreased ROS levels and elevated CAT and POD activities in leaves (Yaghoubian et al., 2014; Swetha and Padmavathi, 2020; Liu et al., 2021). *Si* inoculation also changes the expression of stressed genes under DS. *Si* inoculation, for example, boosted the expression of four drought-associated genes in leaves of drought-stressed cabbage, namely, *DREB2A, CBL1, ANAC072*, and *RD29A* (Sun et al., 2010). However, *Si* inoculation in wheat inhibited CAT activity under drought conditions, achieving a significant level at −0.5 MPa (Hosseini et al., 2017). In maize, CAT and ascorbate peroxidase (APX) activities were also decreased under DS by *Si* (Hosseini et al., 2018). These conflicting results show that *Si* is variable in modulating antioxidant defense systems in host plants and more research needs to be investigated, especially as the molecular mechanism lags behind physiological advances.

Citrus plants, particularly trifoliate orange, have been demonstrated to be a host plant for *Si*, and inoculation with *Si* promoted their growth behavior through increasing auxin levels and nutrient acquisition (Yang et al., 2021a; Liu et al., 2023). However, it is unknown whether and how *Si* impacts the drought tolerance of trifoliate orange in terms of antioxidant defense systems. This study was carried out to investigate the effects of *Si* inoculation on growth, leaf gas exchange, ROS levels, antioxidant enzyme activities, antioxidant levels, and the expression of genes encoding antioxidant enzymes and fatty acid desaturases under DS. Such study can evaluate the potential of *Si* as a biostimulant for drought tolerance in citrus.

## Materials and methods

### Plant culture and experimental design

Four-leaf-old trifoliate orange seedlings grown in autoclaved sands were chosen. *Si* was inoculated at the time of transplanting. *Si* was provided by Prof. Z.-H. Tian (Yangtze University), which was kept in our laboratory. The proliferation of this fungus was performed *in vitro* as per the protocol of Yang et al. (2021a), achieving a spore suspension of 5.0 × 10^8^ CFU/mL and a mycelial solution of 0.018 g/mL.

Three seedlings were planted in a plastic pot that had been pre-filled with an autoclaved mixture consisting of soil and river sands mixed in a 4: 1 ratio by volume to obtain a relative low Olsen-P level (9.73 mg/kg). At the time of transplanting, 12.5 mL of spore suspension and 14.5 mL of mycelial solution were inoculated around roots of potted seedlings as the inoculation treatment (+*Si*). In contrast, the uninoculated treatment (−*Si*) also received the same volume but autoclaved spore suspension and mycelium solution (Rong et al., 2022). The treated seedlings were subjected to the controlled environments described by Cao et al. (2023). The weighing method was used to keep the soil moisture of these potted plants at 75% of the maximum water holding capacity (MWHC) in the field (well-watered, WW). The condition lasted for 7 weeks. Subsequently, the soil moisture regime was altered for half of the plants to 55% of the MWHC in the field (DS) for 9 weeks, while the soil moisture regime remained unchanged for the remaining plants.

Thus, this study consisted of two factors: *Si* inoculation treatments (+*Si* and − *Si*) and two soil moistures (WW and DS). There were four treatments, each with six replications, with a total of 72 seedlings and 24 pots.

### Determination of growth and root fungal colonization frequency

After 9 weeks of drought exposure, the treated plants were harvested and weighed promptly. The Epson Root Scanner (V700) and WinRHIZO software (2007b) were used to quantify root surface area and volume. Then, 1-cm root segments were selected and stained for *Si* colonization in the roots using the method of Phillips and Hayman (1970). In addition, root segments were cut into thin slices of longitudinal sections using a double-sided blade. Subsequently, a drop of 0.05% trypan blue was introduced to observe the fungal colonization. Root fungal colonization was examined under a microscope, and root fungal colonization frequency was estimated as the percentage of *Si* -colonized root segment number to total detected root segment number.

### Determination of leaf physiological variables

On a sunny day (9:00 a.m.) before harvest, leaf gas exchange parameters, including net photosynthetic rate (Pn), transpiration rate (Tr), and stomatal conductance (Gs), were measured on the fourth leaf below the tip of trifoliate orange seedlings using a Li-6400 portable photosynthesizer (Li-COR, USA). The photosynthesizer was preheated for 20 min before used. After calibrating and zeroing the photosynthesizer, the leaf area, ambient water vapor pressure, and CO_2_ concentrations were set at 6.5 cm^2^, 1.01 kPa, and 400 µmol/m^2^/s, respectively. During measurement, the data were recorded after stabilization. Water use efficiency (WUE) was defined as the Pn/Tr ratio.

A portable plant polyphenol-chlorophyll meter (Dualex Scientific+, Orsay, France) was used to measure nitrogen balance index (Nbi) and chlorophyll index (Chi) in leaves.

The concentration of leaf H_2_O_2_ was determined according to the KI colorimetric method reported by Velikova et al. (2000). Leaf ${\text{O}}_{2}^{\cdot -}$ levels were assayed using the protocol outlined by Zou et al. (2015). Leaf MDA concentrations were measured according to the thiobarbituric acid method described by Sudhakar et al. (2001).

Leaf CAT activity was determined colorimetrically at 240 nm according to the method described by He et al. (2020). The absorbance of reaction solutions changed by 0.01 at 240 nm in 1 min as a unit (U) of CAT. Leaf POD activity was assayed using the guaiacol method described by Chance and Maehly (1955), where the absorbance of reaction solutions changed by 0.1 at 470 nm in 1 min as a U of POD. Leaf APX activity was determined as per the protocol outlined by Wu (2018), where the reaction solution consisted of 50 mM potassium phosphate buffer (the enzyme extraction solution), 6 mM AsA, and supernatants. The absorbance of reaction solutions changed by 0.01 at 290 nm in 1 min as a U of APX. Leaf glutathione reductase (GR) activity was analyzed according to the method of Chen and Wang (2002), where the reaction mixture consisted of 1 mM NADPH, 0.1 M tricine-NaOH buffer (the enzyme extraction solution), supernatants, and 5 mM oxidized GSH. The absorbance of reaction solutions changed by 0.01 at 340 nm in 1 min as a U of GR.

Fresh leaf samples (0.30 g) were ground into a homogenate in 5 mL of 5% trichloroacetic acid solution and centrifuged at 15,000×*g* for 15 min. The supernatant was used for the assay of AsA and GSH, and the procedure for the assay had been described in detail by Li et al. (2022).

### Determination of the expression of genes encoding antioxidant enzymes and fatty acid desaturases

Total RNA of leaves was extracted using the TaKaRa MiniBEST Plant RNA Extraction Kit. Following detection of total RNA concentration and purity, total RNA was reverse transcribed into cDNA based on the PrimeScript™ RT reagent Kit with the gDNA Eraser kit. Each treatment’s cDNA obtained was employed as the template for RT-PCR amplification. According to the findings of Wu et al. (2019a), five antioxidant enzyme genes and four fatty acid saturase genes were chosen and thus designed for their primers in qRT-PCR (**Supplementary T** **able S1**). The internal reference gene in this investigation was *β-actin*. The SYBR Green PCR Master Mix and Real-time PCR Detection System (BIO-RAD, Hercules, USA) were used for real-time PCR. There were three biological replicates for each determination. The 2^−ΔΔCt^ method (Livak and Schmittgen, 2001) was used to calculate expression of genes. Relative expression of genes was normalized with the uninoculation treatment under WW conditions.

### Statistical analysis

The two-way analysis of variance under the condition of SAS software (v8.1) was used to compare the variance of the experimental data, and Duncan’s multiple-range test was performed to assess significant (*p*< 0.05) differences across treatments.

## Results

### Effects of DS on root fungal colonization frequency

**Figure 1:**
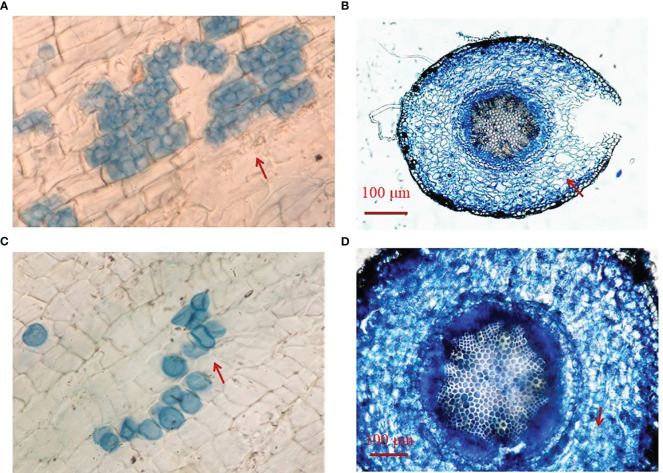
Root colonization of *Serendipita indica* (*Si*) in trifoliate orange seedlings under drought stress **(A, B)** and well-watered **(C, D)** conditions. The red arrow indicates transparent pear-shaped chlamydospores.

Fungal colonization was found in roots of *Si*-inoculated seedlings, but not in no-*Si*-inoculated seedlings, with more transparent pear-shaped chlamydospores in *Si*-inoculated roots under DS (**Figures 1A, B**) than under WW (**Figures 1C, D**). Root fungal colonization frequency was 30.1% under WW conditions and 61.9% under DS conditions, respectively (**Table 1**). *Si* inoculation and DS treatment interacted (*p*< 0.01) to affect root fungal colonization frequency.

**Table 1:** Changes in root fungal colonization frequency and plant growth of trifoliate orange seedlings inoculated with *Serendipita indica* (*Si*) under well-watered (WW) and drought stress (DS) conditions.

Treatments	Root fungal colonization frequency (%)	Plant height (cm)	Leaf number (num./plant)	Biomass (g FW/plant)	Root surface area (cm^2^)	Root volume (cm^3^)	Shoot/root ratio
WW-*Si*	0c	12.98 ± 0.44b	16.72 ± 0.68bc	4.85 ± 0.27b	54.35 ± 2.77b	0.64 ± 0.06b	0.52 ± 0.04b
WW+*Si*	30.1 ± 4.3b	15.83 ± 3.49a	18.44 ± 1.53a	5.94 ± 0.36a	63.76 ± 3.31a	0.72 ± 0.06a	0.65 ± 0.06a
DS-*Si*	0c	12.26 ± 0.31b	15.28 ± 0.98c	3.98 ± 0.17c	42.55 ± 3.90c	0.55 ± 0.03c	0.53 ± 0.04b
DS+*Si*	61.9 ± 5.9a	14.69 ± 2.46ab	18.00 ± 1.55ab	4.53 ± 0.26b	54.01 ± 1.30b	0.63 ± 0.05b	0.64 ± 0.06a
*Significance*							
DS	**	NS	NS	**	**	**	NS
*Si*	**	**	**	**	**	**	**
Interaction	**	NS	NS	*	NS	NS	NS

Data (means ± SD, n = 6) followed by different letters in the same column indicate significant (*p*< 0.05) differences. NS, not significant at *p*< 0.05; *, *p*< 0.05; **, *p*< 0.01.

### Effects of Si inoculation on plant growth variables under DS

DS and Si inoculation significantly impacted plant growth behavior of trifoliate orange seedlings (**Supplementary Figure S1**). DS treatment inhibited plant biomass, root surface area, and root volume of *Si*-inoculated plants by 23.74%, 15.29%, and 12.50%, respectively, compared with WW treatment (**Table 1**). Leaf number, biomass, root surface, and root volume of no-*Si*-inoculated plants were also decreased under DS versus WW by 8.38%, 17.94%, 21.71%, and 14.06%, respectively. *Si* inoculation significantly increased shoot/root ratio, leaf number, biomass, root surface area, and root volume under WW conditions by 24.68%, 17.80%, 22.47%, 26.93%, and 12.50%, respectively, and under DS conditions by 21.78%, 19.82%, 10.29%, 13.82%, 17.31%, and 14.55%, respectively, compared with no-*Si* inoculation. The interaction of DS treatment and *Si* inoculation significantly affected biomass.

### Effects of Si inoculation on leaf gas exchange under DS

**Figure 2:**
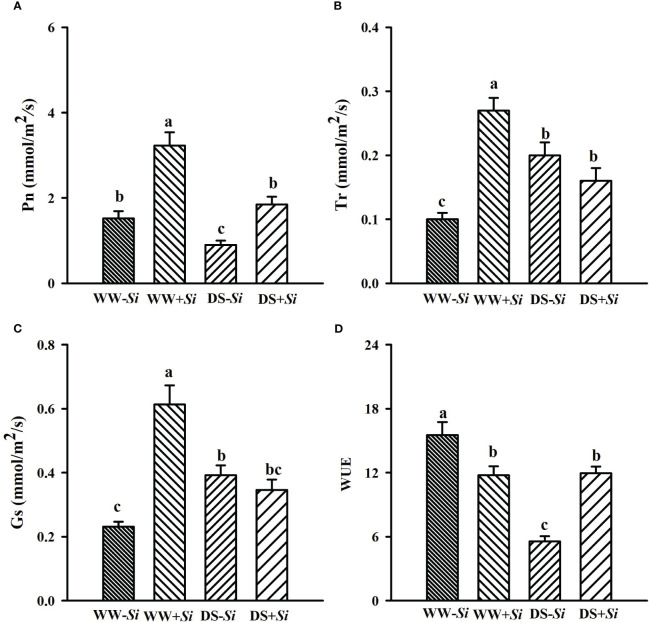
Effects of *Serendipita indica* (*Si*) on leaf net photosynthetic rate (Pn) **(A)**, transpiration rate (Tr) **(B)**, stomatal conductance (Gs) **(C)**, and water use efficiency (WUE) **(D)** of trifoliate orange seedlings under well-watered (WW) and drought stress (DS) conditions. Data (means ± SD, *n* = 4) followed by different letters upon the bars indicate significant (*p<* 0.05) differences.

Leaf Pn, Gs, and Tr in *Si*-inoculated seedlings was inhibited under DS versus WW by 42.89%, 43.63%, and 43.40%, respectively, and leaf Pn and WUE in no-*Si*-inoculated seedlings were also suppressed by 40.86% and 64.20%, respectively (**Figures 2A–D**). Compared with no-*Si* inoculation, *Si* inoculation profoundly raised leaf Pn, Tr, and Gs under WW conditions by 113.01%, 179.16%, and 165.67%, respectively, and it also raised leaf Pn and WUE by 105.71% and 115.29% under DS conditions, respectively. DS and *Si* inoculation interactively (*p*< 0.01) affected Tr, Gs, and WUE (**Table 2**).

**Table 2:** Significance of variables in trifoliate orange seedlings inoculated with *Serendipita indica* (*Si*) under drought stress (DS) conditions.

Variables	DS	*Si*	Interaction	Variables	DS	*Si*	Interaction
Chi	*	**	NS	POD	*	**	**
Nbi	NS	**	**	GR	NS	*	NS
Pn	**	**	NS	APX	**	**	**
Tr	NS	**	**	*PtMn-SOD*	**	**	**
WUE	**	**	**	*PtFe-SOD*	**	**	**
Gs	NS	**	**	*PtCu/Zn-SOD*	**	**	**
H_2_O_2_	**	**	NS	*PtCAT1*	**	**	NS
${\text{O}}_{2}^{\cdot -}$	**	**	**	*PtPOD*	**	**	**
MDA	**	**	NS	*PtFAD2*	**	**	NS
AsA	**	**	**	*PtFAD6*	**	**	**
GSH	**	**	NS	*PtΔ9*	**	**	**
CAT	**	**	NS	*PtΔ15*	**	**	**

NS, not significant at *p*< 0.05; *, *p*< 0.05; **, *p*< 0.01.

### Effects of Si inoculation on leaf chlorophyll index and nitrogen balance index under DS

**Figure 3:**
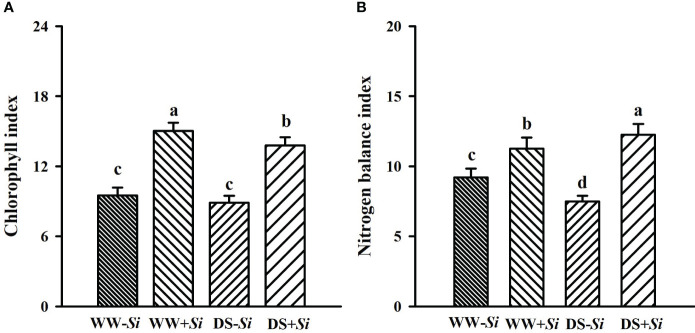
Effects of *Serendipita indica* (*Si*) on leaf chlorophyll index **(A)** and nitrogen balance index **(B)** in leaves of trifoliate orange seedlings under well-watered (WW) and drought stress (DS) conditions. Data (means ± SD, *n* = 4) followed by different letters upon the bars indicate significant (*p<* 0.05) differences.

Compared with WW treatment, soil drought significantly reduced leaf Chi of *Si*-inoculated seedlings and Nbi of no-*Si*-inoculated seedlings by 8.50% and 18.78%, respectively, coupled with an 8.78% significant increase in Nbi of *Si*-inoculated seedlings (**Figures 3A, B**). *Si* inoculation raised leaf Chi and Nbi by 58.33% and 22.34% under WW conditions and 55.34% and 63.84% under DS conditions, respectively, compared with no-*Si* treatment. A significant (*p*< 0.01) interaction appeared in Nbi (**Table 2**).

### Effects of Si inoculation on leaf ROS levels under DS

**Figure 4:**
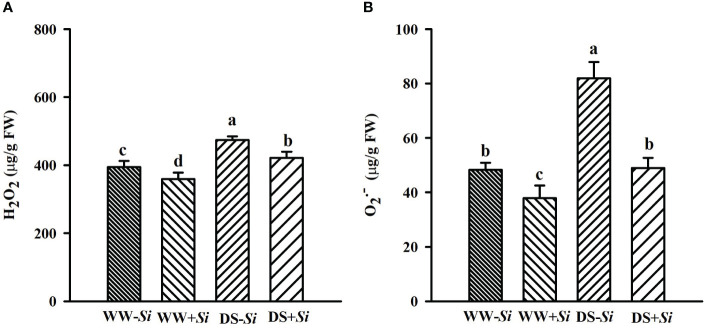
Effects of *Serendipita indica* (*Si*) on leaf hydrogen peroxide (H_2_O_2_) **(A)** and superoxide anion radical ( ${\text{O}}_{2}^{\cdot -}$ ) **(B)** concentrations in trifoliate orange seedlings under well-watered (WW) and drought stress (DS) conditions. Data (means ± SD, *n* = 4) followed by different letters upon the bars indicate significant (*p<* 0.05) differences.

Leaf H_2_O_2_ and ${\text{O}}_{2}^{\cdot -}$ levels were significantly raised under DS versus WW conditions: 17.21% and 29.26% higher in *Si*-inoculated seedlings and 20.21% and 69.66% higher in no-*Si*-inoculated seedlings, respectively (**Figures 4A, B**). Compared with no-*Si* treatment, *Si* inoculation had a significantly inhibitory effect on leaf H_2_O_2_ and ${\text{O}}_{2}^{\cdot -}$ levels, with 8.88% and 21.54% lower under WW and 11.15% and 40.22% lower under DS, respectively. A significant (*p<* 0.01) interaction appeared in ${\text{O}}_{2}^{\cdot -}$ levels (**Table 2**).

### Effects of Si inoculation on leaf MDA levels under DS

**Figure 5:**
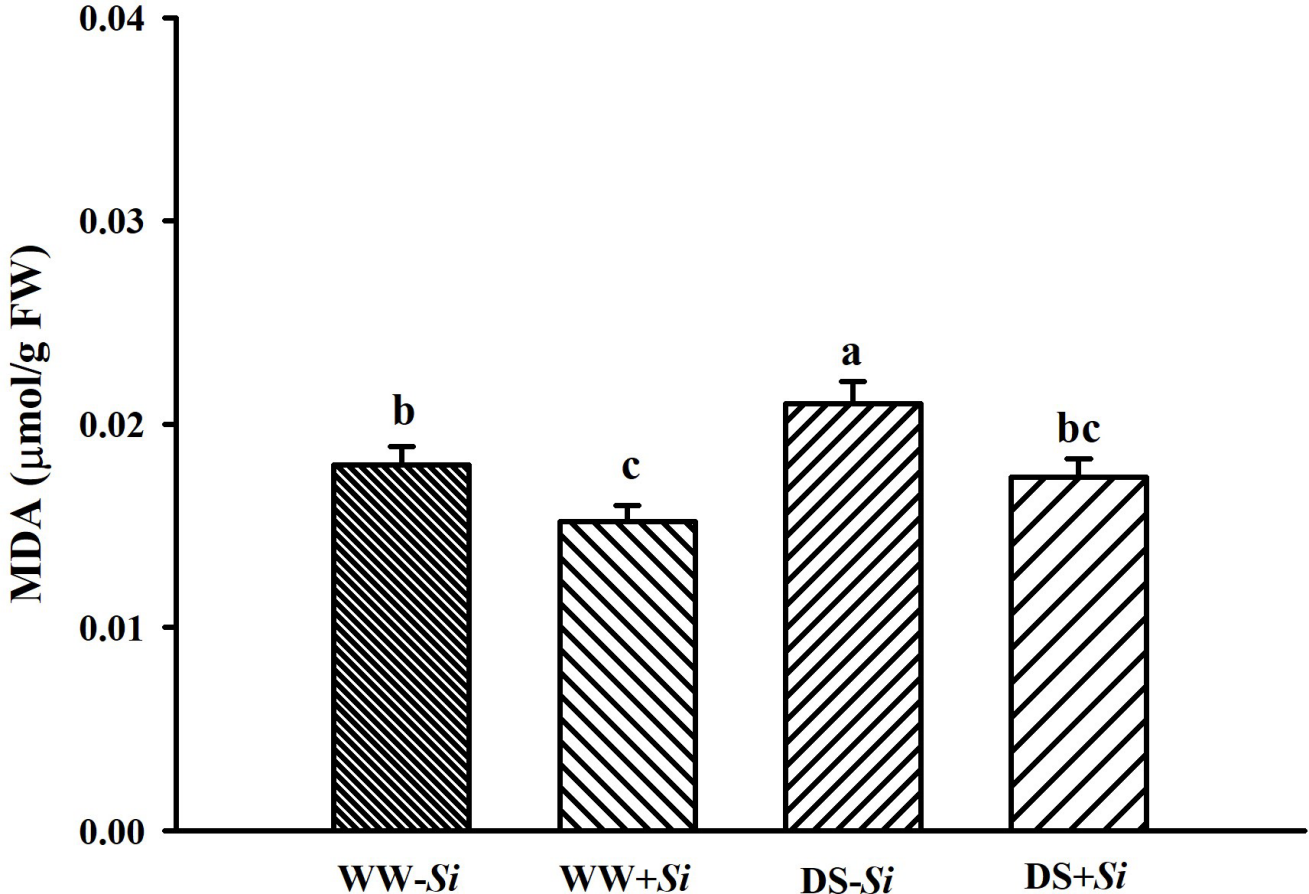
Effects of *Serendipita indica* (*Si*) on leaf malondialdehyde (MDA) concentrations in trifoliate orange seedlings under well-watered (WW) and drought stress (DS) conditions. Data (means ± SD, *n* = 4) followed by different letters upon the bars indicate significant (*p<* 0.05) differences.

Leaf MDA levels were significantly increased by 16.95% in no-*Si*-inoculated seedlings, but not *Si*-inoculated seedlings, under DS versus WW (**Figure 5**). Compared to no-*Si* treatment, *Si* inoculation significantly reduced leaf MDA levels by 15.13% under WW and 17.12% under DS, respectively.

### Effects of Si inoculation on leaf antioxidant levels under DS

**Figure 6:**
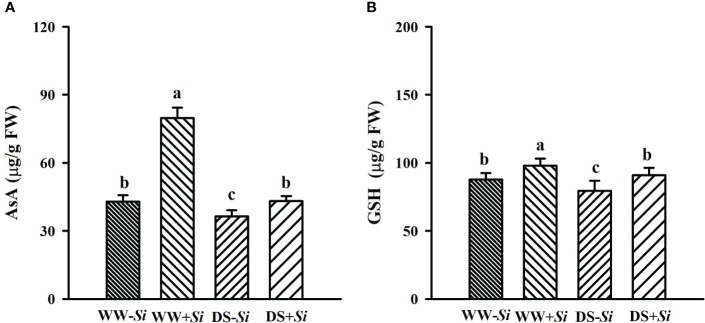
Effects of *Serendipita indica* (*Si*) on ascorbic acid (AsA) **(A)** and glutathione (GSH) **(B)** concentrations in leaves of trifoliate orange seedlings under well-watered (WW) and drought stress (DS) conditions. Data (means ± SD, *n* = 4) followed by different letters upon the bars indicate significant (*p<* 0.05) differences.

Leaf AsA and GSH levels were significantly decreased under DS versus WW conditions by 45.89% and 7.13% in *Si*-inoculated seedlings and 15.04% and 9.42% in no-*Si*-inoculated seedlings, respectively (**Figures 6A, B**). However, *Si* inoculation significantly raised leaf AsA and GSH levels by 85.66% and 11.50% under WW conditions and 18.24% and 14.31% under DS conditions, respectively, compared with no-*Si* inoculation treatment.

### Effects of Si inoculation on leaf antioxidant enzyme activities under DS

**Figure 7:**
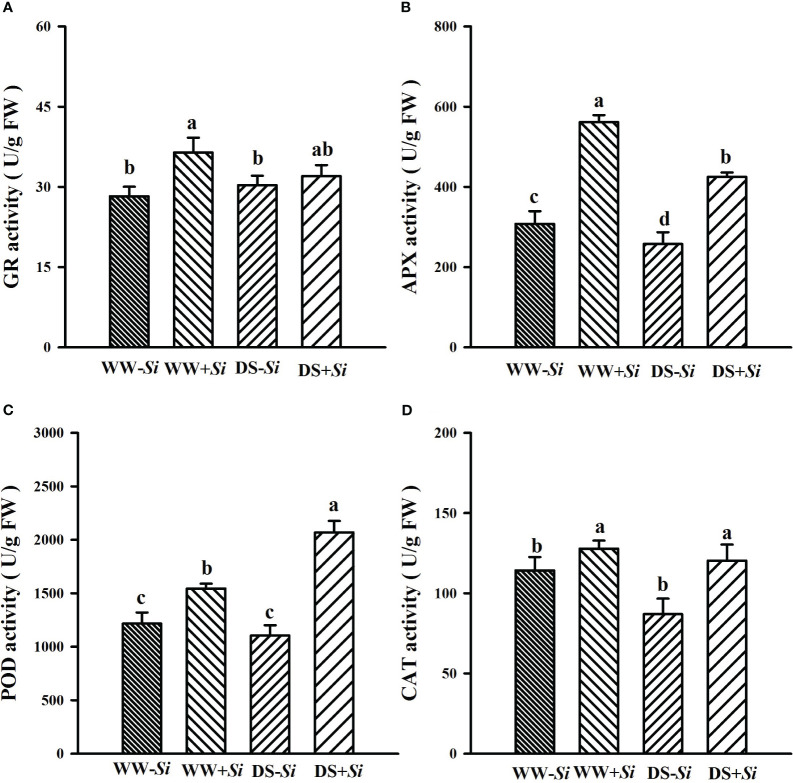
Effects of *Serendipita indica* (*Si*) on glutathione reductase (GR) **(A)**, ascorbate peroxidase (APX) **(B)**, peroxidase (POD) **(C)**, and catalase (CAT) **(D)** in leaves of trifoliate orange seedlings under well-watered (WW) and drought stress (DS) conditions. Data (means ± SD, *n* = 4) followed by different letters upon the bars indicate significant (*p<* 0.05) differences.

Compared with WW treatment, DS treatment significantly decreased leaf GR and APX activities by 12.09% and 24.26% in *Si*-inoculated seedlings, while it distinctly raised leaf POD activities by 34.07% in *Si*-inoculated seedlings, along with a significant decrease in leaf APX and CAT levels by 16.26% and 23.90% in no-*Si*-inoculated seedlings (**Figures 7A–D**). *Si* inoculation significantly increased leaf GR, APX, POD, and CAT activities under WW by 28.98%, 82.34%, 26.84%, and 11.88%, respectively, compared with no-*Si* inoculation. Under DS, *Si* inoculation significantly raised leaf POD, APX, and CAT activities by 87.36%, 64.92%, and 38.43%, respectively, compared with no-*Si* inoculation. A significant (*p*< 0.01) interaction appeared in POD and APX activities (**Table 2**).

### Effects of Si inoculation on leaf antioxidant enzyme genes expression under DS

**Figure 8:**
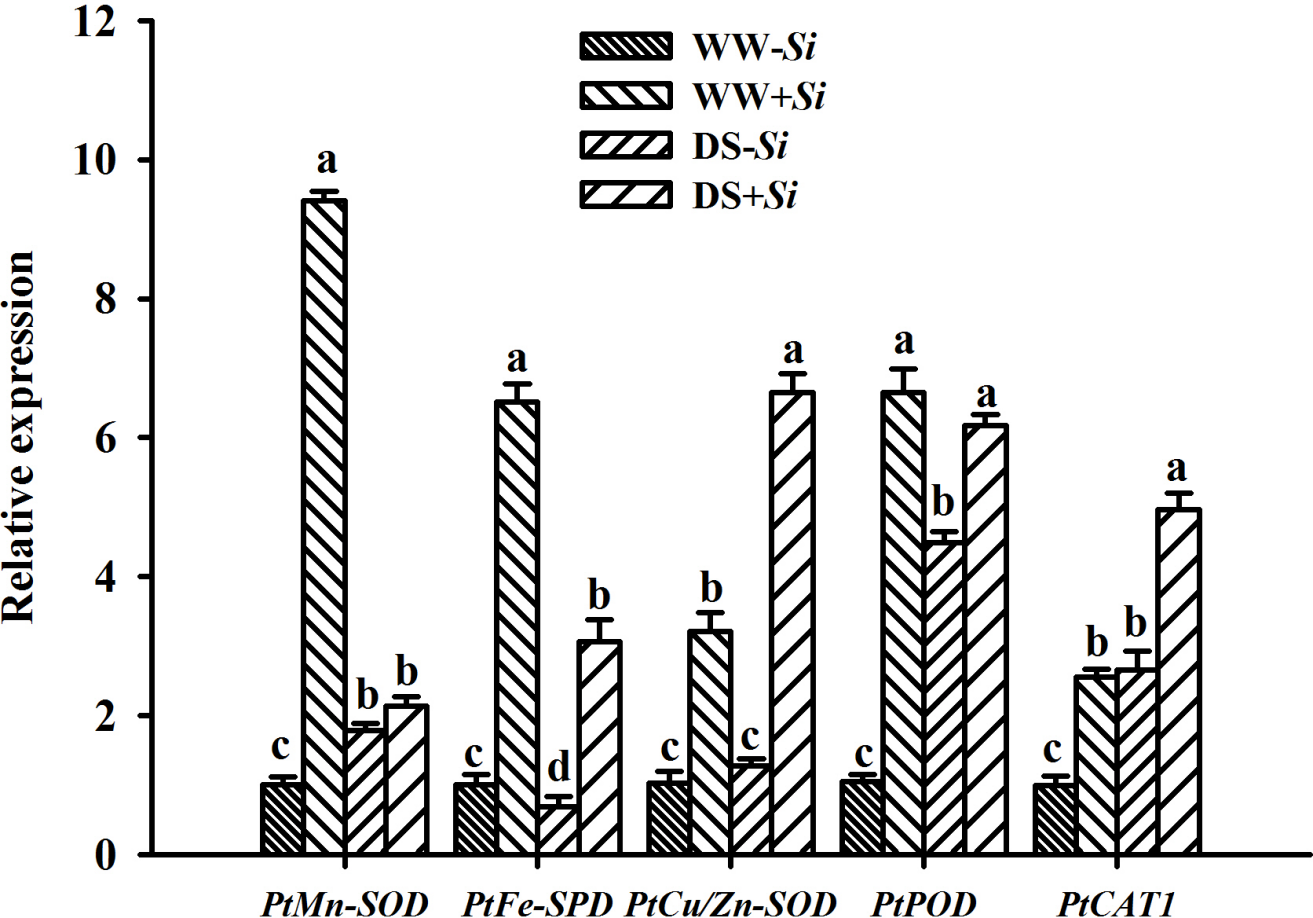
Effects of *Serendipita indica (Si)* on relative expression of five antioxidant enzyme genes in leaves of trifoliate orange seedlings under well-watered (WW) and drought stress (DS) conditions. Data (means ± SD, *n* = 3) followed by different letters upon the bars indicate significant (*p*< 0.05) differences.

Compared with WW treatment, DS treatment triggered upregulation of *PtCu/Zn-SOD* and *PtCAT1* gene expression in leaves of *Si*-inoculated seedlings by 1.07- and 0.94-fold, respectively, but it also suppressed the expression of *PtMn-SOD*, *PtFe-SOD*, and *PtPOD* genes in leaves of no-*Si*-inoculated seedlings by 0.77-, 0.53-, and 0.07-fold, respectively (**Figure 8**). The expression of *PtMn-SOD*, *PtCu/Zn-SOD*, *PtPOD*, and *PtCAT1* genes in leaves of no-*Si* inoculated seedlings was upregulated under DS versus WW conditions by 0.79-, 0.25-, 3.24-, and 1.65-fold, respectively, accompanied by the downregulated expression of *PtFe-SOD* genes. *Si* inoculation induced the upregulated expression of *PtMn-SOD*, *PtFe-SOD*, *PtCu/Zn-SOD*, *PtPOD*, and *PtCAT1* genes under WW conditions by 8.38-, 5.46-, 2.12-, 5.28-, and 1.56-fold, respectively, compared with no-*Si* inoculation. Under DS conditions, *Si* inoculation upregulated the expression of *PtMn-SOD*, *PtFe-SOD*, *PtCu/Zn-SOD*, *PtPOD*, and *PtCAT1* genes by 0.19-, 3.43-, 4.19-, 0.38-, 0.87-fold, respectively. There was a significant interaction between DS treatment and *Si* inoculation on the expression of leaf *PtMn-SOD*, *PtFe-SOD*, *PtCu/Zn-SOD*, and *PtPOD* genes (**Table 2**).

### Effects of Si inoculation on leaf fatty acid desaturase genes expression under DS

**Figure 9:**
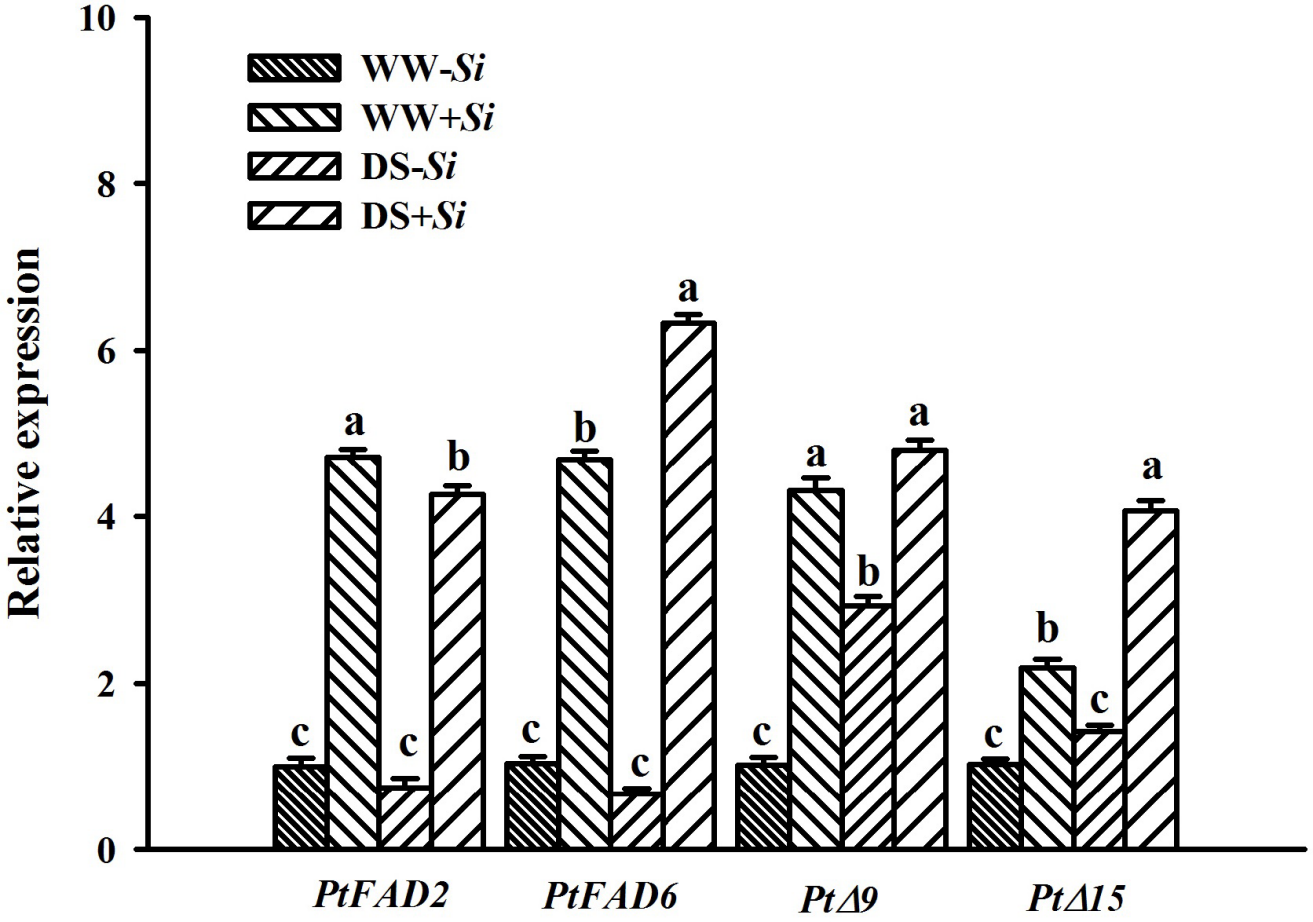
Effects of *Serendipita indica (Si)* on relative expression of four fatty acid desaturase genes in leaves of trifoliate orange seedlings under well-watered (WW) and drought stress (DS) conditions. Data (means ± SD, *n* = 3) followed by different letters upon the bars indicate significant (*p*< 0.05) differences.

The expression of *PtFAD2* gene in leaves of *Si*-inoculated plants was downregulated by 0.10-fold under DS versus WW conditions, accompanied by 0.35- and 0.86-fold upregulation of *PtFAD6* and *PtΔ15*, respectively (**Figure 9**). In leaves of no-*Si*-inoculated plants, the expression of *PtΔ9* gene was upregulated by 1.94- fold under DS versus WW conditions. Compared with no-*Si*-inoculated treatment, *Si* inoculation significantly raised the expression of leaf *PtFAD2*, *PtFAD6*, *PtΔ9*, and *PtΔ15* genes by 3.70-, 3.65-, 3.30-, and 1.18-fold under WW conditions, respectively, and by 4.94-, 8.52-, 0.63-, and 1.86-fold under DS conditions, respectively. DS treatment and *Si* inoculation interacted significantly to affect the expression of *PtFAD6*, *PtΔ9*, and *PtΔ15* genes (**Table 2**).

## Discussion

In this study, root colonization frequency of *Si* in trifoliate orange seedlings was significantly increased under DS versus WW conditions, which is consistent with *Si*-colonized white clover under DS (Rong et al., 2022). *Si* was isolated from arid zones and is therefore well adapted to drought (Boorboori and Zhang, 2022). It has been shown that *Si* preferentially colonized the root-hair zone, and the colonization frequency of *Si* increased with root senescence (e.g., under drought conditions) (Schäfer and Kogel, 2009). Nevertheless, a decrease in root *Si* colonization was observed in wheat plants under DS versus WW conditions (Yaghoubian et al., 2014). In *Eleusin coracana* plants, DS also induced the decrease in root *Si* colonization (Tyagi et al., 2017). In maize, root *Si* colonization was not distinctly affected by DS (Xu et al., 2017). This suggests that the response of root *Si* colonization to DS is variable.

Plants change root architecture in response to DS, with reduced lateral root density and allocating more nutrients to old roots (Lynch, 2018). Soil drought can strongly inhibit crop growth (Wahab et al., 2022). The present study also observed a decrease in plant growth variables under DS versus WW conditions, regardless of *Si* inoculation or not. However, *Si*-inoculated trifoliate orange seedlings represented greater plant growth performance and root surface area and volume, regardless of WW and DS. Similar results were reported in barley and wheat inoculated with *Si* under DS conditions (Hosseini et al., 2017; Ghaffari et al., 2019). Such changes may be linked to the fact that *Si* could promote the auxin and cytokinin synthesis of host plants (Liu et al., 2023; Rong et al., 2023).

Leaf gas exchange is closely linked to growth responses (Wu et al., 2019b). In the present study, DS treatment significantly reduced Pn, WUE, and Nbi in leaves of no-*Si*-inoculated seedlings, and Pn, Tr, Gs, Chi, and Nbi in *Si*-inoculated seedlings, compared with WW treatment. Interestingly, DS significantly raised Tr and Gs in no-*Si*-inoculated seedlings compared with WW treatment. This may be explained by the fact that prolonged DS irreversibly damages leaf tissues of no-*Si*-inoculated plants, thus accelerating Tr and Gs and leaving them in a more drought state (Yang et al., 2021b). In addition, *Si* application considerably raised Pn, Gs, Tr, Chi, and Nbi in WW-treated seedlings and Pn, Chi, Nbi, and WUE in DS-treated seedlings, compared with no-*Si-*inoculated treatment. This showed a significant improvement of WUE in *Si*-inoculated seedlings only under DS conditions, which was related to the involvement of mycelium of *Si* in water uptake. On the other hand, the *Si* inoculation also enhanced Pn by promoting chlorophyll formation, accompanied by an enhancement of Nbi. Under DS, *Si*-inoculated rice plants also exhibited similar results (Saddique et al., 2018). In *Eleusine coracana* plants, *Si* inoculation distinctly raised chlorophyll levels under DS (Tyagi et al., 2017). These increases under both *Si* inoculation and DS conditions are associated with *Si*-promoted P uptake and photosystem II efficiency (Tariq et al., 2017). Li et al. (2021) also observed the raised Chi level in *Ipomoea batatas* plants after *Si* inoculation. Proteomics analysis showed that *Si* inoculation on barley led to significant upregulation of various photosynthesis-related protein levels under DS, including photosystem complex proteins and photorespiratory enzymes (Ghaffari et al., 2019).

In the present study, ROS levels were induced to increase, and MDA was elevated in *Si*- and no-*Si-*inoculated trifoliate orange seedlings under DS versus WW conditions, indicating that the drought triggered oxidative damage in trifoliate orange seedlings. Furthermore, inoculation with *Si* was able to significantly reduce leaf H_2_O_2_ and ${\text{O}}_{2}^{\cdot -}$ levels as well as MDA concentrations, accompanied by a higher decrease under DS conditions than under WW conditions. Similar result was reported in maize plants under DS after *Si* inoculation (Kaboosi et al., 2023). MDA is a by-product of membrane lipid damage under DS (Pavlović et al., 2018). Sun et al. (2010) found that MDA levels in leaves of *Si*-colonized *B. campestris* plants in response to DS were delayed, coupled with the upregulation of antioxidant enzyme activities within 24 h. *Si* inoculation under DS also triggered a decrease in leaf ROS and MDA contents in *Triticum aestivum* and *Solanum melongena* (Yaghoubian et al., 2014; Swetha and Padmavathi, 2020). Therefore, *Si*-inoculated plants recorded lower oxidative burst and oxidative damage under drought, showing their enhanced drought tolerance. Nevertheless, *Si* colonization in walnut plants dramatically decreased MDA levels in leaves, but not roots under DS, suggesting a tissue dependency (Liu et al., 2021).

In plants, the AsA-GSH cycle, mediated by GR and APX, is associated with H_2_O_2_ scavenging (Irshad et al., 2021). Our study indicated that drought treatment markedly reduced leaf AsA and GSH levels, whereas inoculation with *Si* significantly increased leaf AsA and GSH levels, regardless of soil moisture regimes. Meanwhile, *Si*-inoculated seedlings also maintained a high APX activity under drought and a higher APX and GR activity under WW than no-*Si*-inoculated seedlings. This means that *Si*-inoculated plants have a more efficient AsA-GSH cycle to scavenge ROS under DS, which is in agreement with the results obtained by Rong et al. (2022) inoculating *Si* on white clover under DS. In *A. thaliana*, *Si* inoculation responded to DS by enhancing the AsA-GSH cycle pathway in plants (Sun et al., 2010). Under salt stress conditions, *Si* also provided tomato plants with a superior AsA-GSH cycle to eliminate ROS (Ghorbani et al., 2018), suggesting that *Si* plays an important role in modulating the AsA-GSH cycle under adversity.

This study also represented enhanced CAT and POD activities after *Si* inoculation under WW and DS. It has been demonstrated that *Si* colonization raised antioxidant enzyme activities in host plants including CAT and POD (Lin et al., 2019). Under drought, *Si* inoculation also enhanced the CAT activity of *I. batatas* (Li et al., 2021). Wheat inoculated with *Si* exhibited lower levels of lipid peroxidation as well as higher CAT and APX activities under DS (Yaghoubian et al., 2014). *Si* inoculation, on the other hand, reduced CAT activity of drought-stressed wheat and APX activity of drought-stressed wheat and maize (Hosseini et al., 2017; Hosseini et al., 2018). This indicated that *Si* effects on antioxidant enzyme activities are variable. Alternatively, *Si* inoculation activates drought-escape mechanisms in host plants, thereby doing not require enhanced antioxidant enzyme activities in response to drought (Jangir et al., 2021). In follow-up studies, we should explore how the *Si* activates the signaling pathway of antioxidant enzyme system in host plants subjected to DS.

Inoculation with *Si* also altered the expression of genes encoding antioxidant enzymes and fatty acid desaturases under DS, with increased expression in leaf *PtFe-SOD*, *PtCu/Zn-SOD*, *PtPOD*, *PtCAT1*, *PtFAD2*, *PtFAD6*, *PtΔ9*, and *PtΔ15* genes. Similarly, inoculation of *Rhizophagus irregularis* upregulated leaf *PpGR*, *PpMn-SOD*, and *PpCu/Zn-SOD* expression of *Robinia pseudoacacia* plants under 200 mM NaCl conditions, but not 100 mM NaCl (Chen et al., 2020). Wu et al. (2019a) also reported that *Funneliformis mosseae* inoculation upregulated root *PtFAD2*, *PtFAD6*, and *PtΔ9* gene expression in trifoliate orange under DS. In field citrus, *Si* inoculation also upregulated the expression of *CsPOD*, *CsCAT1*, and *CsFAD6* in leaves (Li et al., 2022). This suggests that even in the absence of abiotic stress, *Si* can activate the expression of antioxidant defense genes in host plants. In leaves of *B. campestris* and maize, *Si* inoculation also upregulated the expression of stressed genes (*DREB2A*, *CBL1*, *ANAC072*, and *RD29A*) under soil drought (Sun et al., 2010; Xu et al., 2017). In *Gerbera jamesonii* seedlings, *Si* inoculation also triggered the upregulated expression of *NHX2* and *SOS1* under salt stress (Chen et al., 2022). Sun et al. (2010) proposed that the Ca^2+^ sensing regulatory protein could activate *Si* to induce drought-responsive gene expression. However, whether this case occurred in this study remains to be verified.

## Conclusions

In summary, *Si* inoculation alleviated the inhibitory effect of soil drought on growth, Pn, WUE, and Chi of trifoliate orange seedlings, as well as the oxidative damage. This study firstly reported that low oxidative burst in *Si*-inoculated seedlings exposed to soil drought was associated with increased antioxidant enzyme activities and antioxidant levels, as well as upregulated expression of genes encoding antioxidant enzymes and fatty acid desaturases. *Si* has a high potential as a biostimulator for enhanced plant drought tolerance.

## Data availability statement

The original contributions presented in the study are included in the article/**Supplementary Material**. Further inquiries can be directed to the corresponding author.

## Author contributions

Conceptualization, Q-SW and J-LC; data curation, J-LC and YW; methodology, J-LC. resources, Q-SW; supervision, Q-SW; writing—original draft, YW; writing—review and editing, AH, EA, and Q-SW. All authors contributed to the article and approved the submitted version.
