# Supplementary material for: Serendipita indica mitigates drought-triggered oxidative burst in trifoliate orange by stimulating antioxidant defense systems
Source: Front Plant Sci. 2023 Oct 4;14:1247342. doi: 10.3389/fpls.2023.1247342 (PMC10582986; doi:10.3389/fpls.2023.1247342)
Supplement: Supplementary file 1 [file DataSheet_1.pdf]

Supplementary material Table S1 Gene-specific primer sequences used in this study

Genes	Gene ID	Forward primer (5′→3′)	Reverse primer (5′→3′)
PtFe-SOD	Cs7g19250	AGTAAGGAGCGGCGAGTA	GTGGCTAATGCGGTGAAT
PtMn-SOD	Cs7g29850	GGCGAGCCACCACATAGT	CACCCTCAGCATTCATCTTTT
PtCu/Zn-SOD	Cs3g12080	GGACCAGCATGGACTACAAGACC	GGATGCCGGTGGAAGTGTTACC
PtPOD	Cs1g18600	GGCTCAACTTGTCCACCTC	TATCGTCGCCCTGTCTG
PtCAT1	Cs3g27280	TAACAGTGGAGGAGCGAACA	GGAGCCAGTGCTAAGGGT
PtFAD2	orange1.1t02241	AGGAGGCAAGAGTGGAGGATAAGG	GGTGCAGGTGGACGAATGTCTG
PtFAD6	Cs8g17450	CTGCACGGAGATACAGCTTGGC	GGAATGTGAGGAGCCGTATGATGC
PtΔ9	orange1.1t03533	TGCCTGCTCACTTGATGTACGATG	CTCCTCCAGCCTTCTGATTCTTGC
PtΔ15	Cs6g08600	CAAGAACTGGTCTAGCAGCCTCAG	ATGTGGCTGGACCTTGTGACTTAC
β-Actin	Cs1g05000	CCGACCGTATGAGCAAGGAAA	TTCCTGTGGACAATGGATGGA

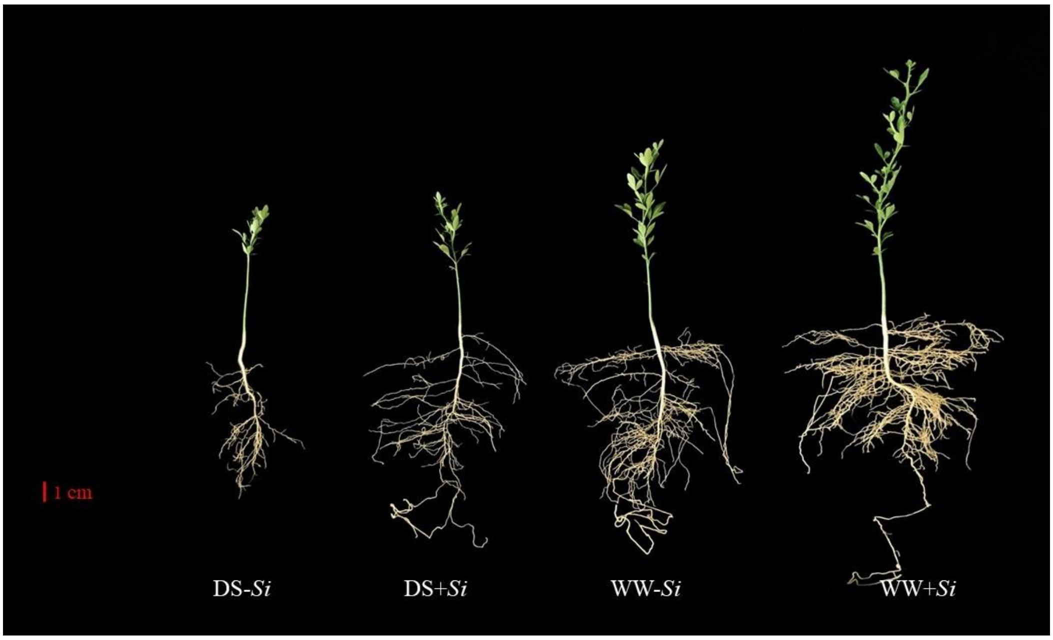

Supplementary material Figure S1 Changes in plant growth of trifoliate orange inoculated with *Serendipita indica* (*Si*) under well-watered (WW) and drought stress (DS) conditions.
